# Effects of Exergaming on Musculoskeletal Pain in Older Adults: Systematic Review and Meta-analysis

**DOI:** 10.2196/42944

**Published:** 2023-04-25

**Authors:** Nan Mo, Jin yu Feng, Hai xia Liu, Xiao yu Chen, Hui Zhang, Hui Zeng

**Affiliations:** 1 Xiangya Nursing School Central South University Changsha China

**Keywords:** aged, exergaming, pain, review, video game, virtual reality

## Abstract

**Background:**

Exercise is effective for musculoskeletal pain. However, physical, social, and environmental factors make it difficult for older adults to persist in exercising. Exergaming is a new pathway that combines exercise with gameplay and may be helpful for older adults to overcome these difficulties and engage in regular exercise.

**Objective:**

This systematic review aimed to determine the efficacy of exergaming to improve musculoskeletal pain in older adults.

**Methods:**

The search was performed in 5 databases (PubMed, Embase, CINAHL, Web of Science, and Cochrane Library). The risk of bias for randomized controlled studies was assessed using the revised Cochrane Risk of Bias tool in randomized trials (RoB 2), and the methodological quality was assessed using the Physiotherapy Evidence-Based Database scale. Standardized mean difference and 95% CI were calculated using fixed-effects model meta-analyses in the Review Manager version 5.3 (RevMan 5.3).

**Results:**

Seven randomized controlled studies were included, which contained 264 older adults. Three of the 7 studies reported significant improvements in pain after the exergaming intervention, but only 1 reported a significant difference between groups after adjustment for baseline (*P*<.05), and another reported a significant improvement in thermal pain between the 2 groups (*P*<.001). The results of the meta-analysis of the 7 studies showed no statistically significant improvement in pain compared to the control group (standardized mean difference –0.22; 95% CI –0.47 to 0.02; *P*=.07).

**Conclusions:**

Although the effects of exergames on musculoskeletal pain in older adults are unknown, exergame training is generally safe, fun, and appealing to older adults. Unsupervised exercise at home is feasible and cost-effective. However, most of the current studies have used commercial exergames, and it is recommended that there should be more cooperation between industries in the future to develop professional rehabilitation exergames that are more suitable for older adults. The sample sizes of the studies included are small, the risk of bias is high, and the results should be interpreted with caution. Further randomized controlled studies with large sample sizes, high quality, and rigor are needed in the future.

**Trial Registration:**

PROSPERO International Prospective Register of Systematic Reviews CRD42022342325; https://www.crd.york.ac.uk/prospero/display_record.php?RecordID=342325

## Introduction

### Background

The International Association for the Study of Pain defines pain as an unpleasant sensory and emotional experience associated with or resembling actual or potential tissue damage [1]. Pain is classified as acute, subacute, and chronic. Fewer than 4 weeks is acute pain, and the presence of 4 weeks to 3 months is subacute pain, and chronic pain is a condition that persists or recurs for more than 3 months [2]. The causes of pain in older individuals are often due to osteoarthritis, postherpetic neuralgia, diabetic neuropathy, spondylosis and radiculopathy, poststroke pain, and Parkinson disease [3]. According to data from the 2016 American Health Interview Survey [4], 20.4% of American adults have chronic pain, with 8% of American adults having a high impact on chronic pain [5]. The prevalence of pain increases with age [6]. Individuals aged 60 years and older are classified as older individuals by the World Health Organization [7]. Data from 1999 to 2019 showed that 57%-61% of community-dwelling older individuals reported intermittent or daily musculoskeletal pain [8]. By 2030, approximately 66% of people over the age of 65 years will have chronic pain globally [9]. Pain affects sleep and mood, increases the risk of falls, and reduces the quality of life [9-11]. It imposes a heavy burden on society [12].

Changes in the efficacy of analgesic drugs occur due to aging, such as a possible weakening in analgesic effect and a decrease in the efficiency of drugs acting on peripheral sensitization [13]. It is worth noting that medications may make older individuals more debilitated and adverse reactions occur more frequently [3]. Nonpharmacological treatments have therefore been used for pain relief. The efficacy of exercise in alleviating pain has been demonstrated [14], and exercising in nonpainful areas of the body has an analgesic effect on painful areas [15]. Older adults with poor physical function prefer to exercise at home at no cost [16]. However, the lack of supervision and motivation at home leads to low exercise adherence [17], which further leads to poorer treatment effects [18,19].

Technology can serve as an effective strategy to confront these challenges. Exergames are video games or virtual reality (VR) games that combine gameplay with physical training and are potential tools to make exercises more enjoyable and increase motivation and compliance for physical activity [20-23]. Results of systematic reviews showed that exergames could improve the activities of daily life [24], cognitive [25] and physical function [26], balance [27], walking speed [28], and depression [29,30] among older adults. There was some evidence of randomized controlled trials (RCTs) supporting the benefits of exergames for improving pain in older adults [31,32].

### Research Gap and Aim

To our knowledge, 3 reviews have systematically summarized the effects of exergames on pain. A systematic review [33] included thirteen clinical studies, and the mean age of participants ranged from 23.9 (SD 6.8) years to 54.9 (SD 11.8) years. The 6 included controlled trials showed that interactive VR exergames may divert attention from pain and alleviate pain postmastectomy and ankylosing spondylitis, but the results were inconsistent for people with neck pain. The remaining 7 uncontrolled studies showed that interactive VR exergames reduced neuropathic limb pain and phantom limb pain, but did not affect nonspecific chronic back pain. A systematic review and meta-analysis [34] also showed that exergames can improve pain perception in females older than 18 years with fibromyalgia. However, a systematic review and meta-analysis [35] that included 7 RCTs concluded that there was insufficient evidence that exergames can improve musculoskeletal pain in the participants and the mean age ranged from 33.5 (SD 9.5) years to 80 years. The results of these reviews are inconsistent, as well as have some limitations. In the first place, they were not focused on older adults. Secondly, the included studies were not all RCTs. In further, some of them did not perform a meta-analysis and the results were not rigorous enough. Therefore, the purpose of this study is to review the efficacy of exergames for musculoskeletal pain in older adults.

## Methods

### Overview and Registration

The report of this systematic review and meta-analysis is consistent with the updated guidelines of the PRISMA (Preferred Reporting Items of Systematic Reviews and Meta-Analyses) 2020 Statement (Multimedia Appendix 1) [36]. The registration number is CRD42022342325.

### Literature Search

A systematic literature search was carried out in 5 databases, PubMed, CINAHL (through EBSCO), the Cochrane Library, Web of Science, and Embase, from the inception to March 4, 2022. The combinations of Medical Subject Headings and free-text terms were used, and concepts included were exergaming, pain, and aged (see Multimedia Appendix 2).

### Eligibility Criteria

The following were the criteria for including the articles: (1) participants’ mean age was more than 60 years and they suffered from musculoskeletal pain; (2) game technology was used to enable participants to exercise; (3) the control group was either active control (other interventions but no gameplay) or passive control (eg, usual care, no treatment, or waiting list); (4) the pain was involved in clinical outcomes; (5) the article had been published in a peer-reviewed publication with a RCT; and (6) the articles were written in English.

Exclusion criteria were as follows: (1) reviews, editorials, conference abstracts, and protocols, or full text was not available; (2) incomplete information on the intervention; (3) outcome data for pain were not statistically analyzed; and (4) duplicate publications or no restrictions on the publication date.

### Study Selection

A researcher searched the 5 databases according to the search strategies. Duplicates were excluded by EndNote 9X. Two researchers independently reviewed the titles and abstracts of records before reading the entire text for rescreening to identify the included literature based on the eligibility criteria. Any differences were settled through discussion or by consulting a third researcher.

### Data Extraction

The data were extracted by 2 independent researchers using the self-developed form in Excel (Microsoft Corp), comprising study characteristics, participant characteristics, intervention details, attrition, supervision, adverse events, experience, measurement tools for the outcome, and the key results. Following that, the 2 researchers subsequently cross-checked. A third investigator was consulted in the event of a dispute.

### Risk of Bias and Methodological Quality Assessment

The included studies’ quality was evaluated using the revised Cochrane Risk of Bias tool in randomized trials (RoB 2) [37]. The risk of bias in 5 domains, including (1) randomization procedure; (2) deviations from intended interventions; (3) missing outcome data; (4) measurement of the outcome; and (5) selection of the reported result, was appraised using 3 degrees of “low risk,” “some concerns,” or “high risk.” When at least one domain was considered to have “some concerns,” but no domain was deemed to have “high risk,” the study was labeled as having “some concerns of bias.” When at least one domain was deemed “high risk” and many domains were deemed “some concerns,” the study was deemed “high risk of bias” [37].

The Physiotherapy Evidence-Based Database scale was used to assess the methodological quality of randomized controlled studies. The scale is a specific instrument for clinical studies of physical therapy interventions [38]. It comprises 11 items related to the selection, detection, performance, information, and attribution bases domains. Research with a score lower than 4 is regarded as bad, 4-5 is considered fair, 6-8 is considered good, and 9-10 is considered excellent [39].

The assessments were conducted independently by 2 independent evaluators. One reviewer resolved the disagreement.

### Data Synthesis

The equation (Mean_change_=Mean_after_–Mean_baseline_) and (SD_change_=√[SD^2^_baseline_+SD^2^_after_–{2×correlation×SD_baseline_×SD_after_}]) were used to calculate the mean change and corresponding SD, and the correlation was set to 0.5. SDs were not given in the study and were obtained by converting the means, sample sizes, and *P* values of the changes in the intervention and control groups [40]. The effect size was measured by the standardized mean difference (SMD) corrected for small sample sizes (Hedges *g*) [40]. Hedges *g* estimates of <0.30, ≥0.30 and <0.60, and ≥0.60 were considered small, moderate, and large, respectively [41]. The heterogeneity among studies was quantified based on the *I*^2^ statistic, with 0%-40% may not be important; 30%-60% may represent moderate heterogeneity; 50%-90% may represent substantial heterogeneity; and 75%-100% may represent considerable heterogeneity [42]. A fixed-effects model was used when *I*^2^≤50%, otherwise a random-effects model was used. We performed subgroup analysis to explore which treatments were more effective or what nature of pain exergaming was more effective for. Meta-analysis was performed using Review Manager version 5.3 (RevMan 5.3). We did not perform a publication bias test because fewer than 10 studies were included [42].

## Results

### Study Selection

**Figure 1:**
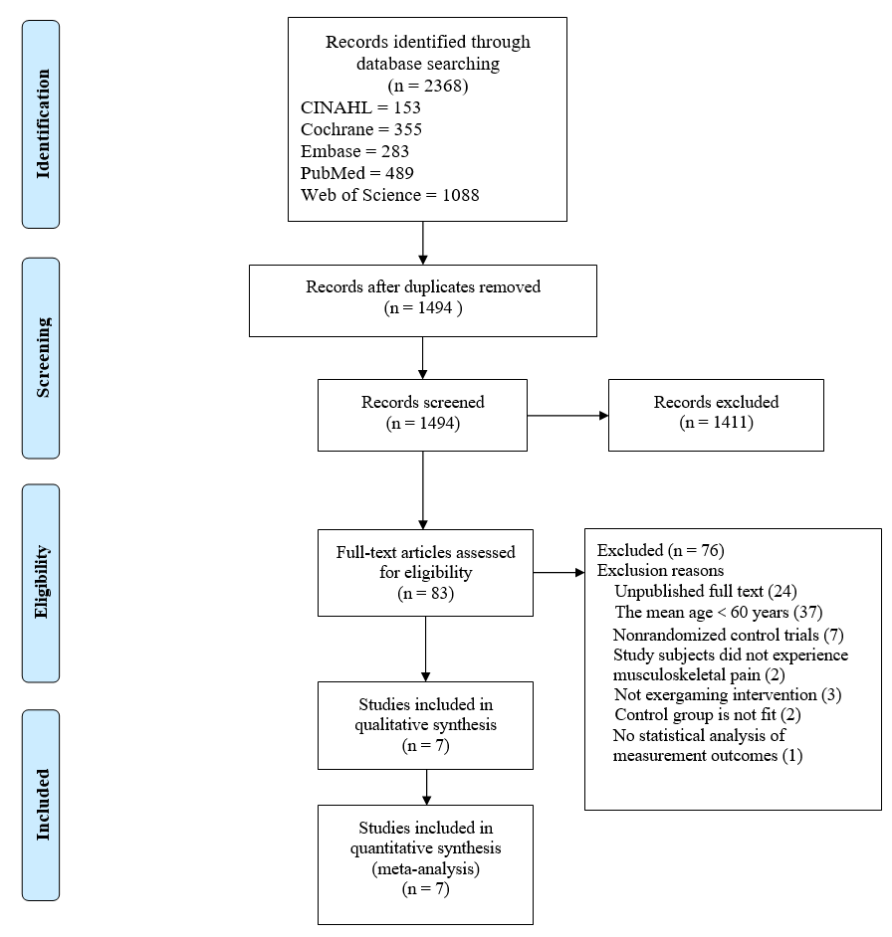
PRISMA (Preferred Reporting Items of Systematic Reviews and Meta-Analyses) flow diagram of study selection according to PRISMA [43].

By searching the databases, 2368 records were found. After removing 874 duplicates, 1494 records were evaluated. A total of 1411 records were excluded according to the eligibility criteria. The remaining 7 studies were analyzed. The PRISMA flow diagram depicts the search and screening procedure (Figure 1).

### Quality Assessment

**Figure 2:**
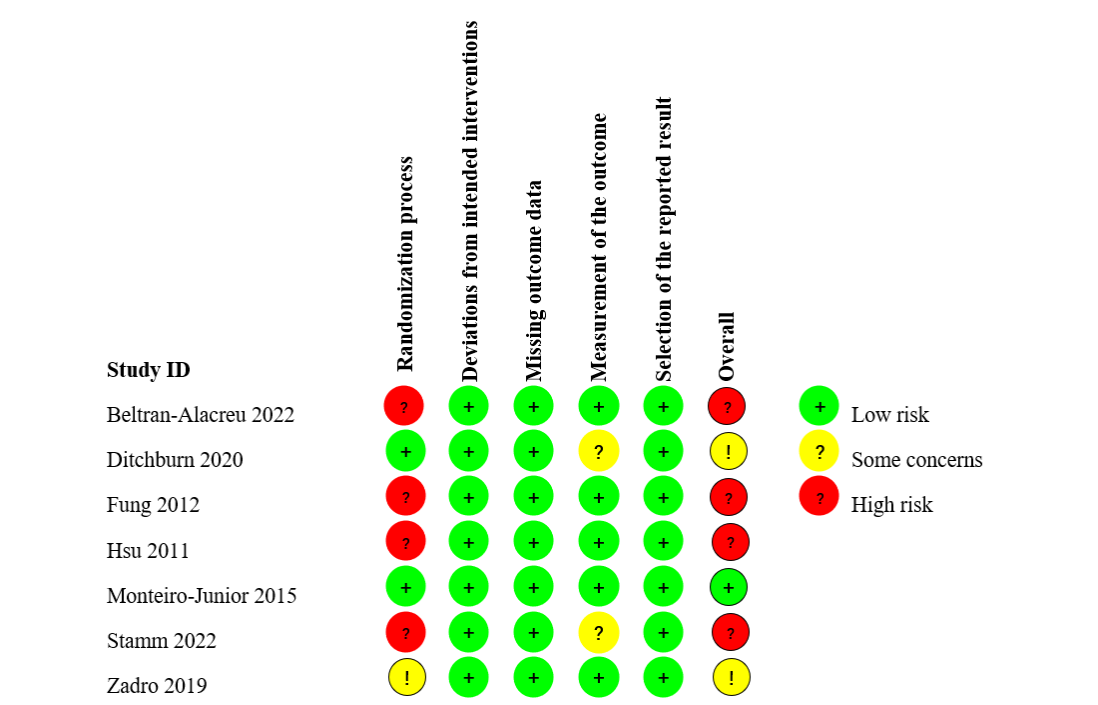
Risk of bias based on revised Cochrane Risk of Bias tool in randomized trials (RoB 2) [37].

**Figure 3:**
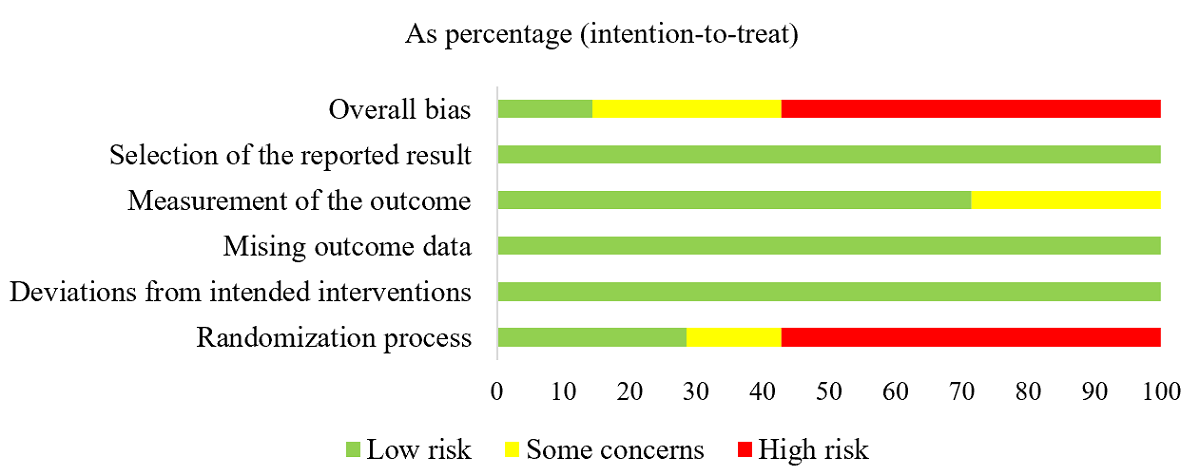
The overall risk of bias in randomized controlled trials.

Figures 2 and 3 summarized the risk of bias assessment for the 7 studies that were included. One study (14%) was classified as “low risk” [44], 2 studies (29%) as “some concerns” [31,32], and 4 studies (57%) as “high risk” [21,22,45,46]. All studies were judged “low risk” for the domains “Deviations from intended interventions,” “Missing outcome data,” and “Selection of the reported result.” Four studies (57%) were assessed as “high risk” for the domain “Randomization procedure” due to no report allocation concealment approaches, while 1 research (14%) was evaluated as “some concerns” related to baseline imbalance. Due to nonblind assessors, 2 studies (29%) were determined to have “some concerns” in the domain “Measurement of the result.”

Physiotherapy Evidence-Based Database scores (mean score approximately 7, range 6-8) demonstrated good overall methodological quality. Monteiro-Junior et al [44] received the highest score of 8, while Stamm et al [46] had the lowest score of 6 (Table 1).

**Table 1:** Methodological quality as assessed by the Physiotherapy Evidence-Based Database Scale. Item 1 did not count toward the total score.

Study, year	1^a^	2^b^	3^c^	4^d^	5^e^	6^f^	7^g^	8^h^	9^i^	10^j^	11^k^	Total score	Methodological quality
Beltran-Alacreu et al [22], 2022	1	1	0	1	0	0	1	1	1	1	1	7	Good
Ditchburn et al [31], 2020	1	1	1	1	0	0	0	1	1	1	1	7	Good
Fung et al [45], 2012	1	1	0	1	0	0	1	1	1	1	1	7	Good
Hsu et al [21], 2011	1	1	0	1	0	0	1	1	1	1	1	7	Good
Monteiro-Junior et al [44], 2015	1	1	1	1	0	0	1	1	1	1	1	8	Good
Stamm et al [46], 2022	1	1	0	1	0	0	0	1	1	1	1	6	Good
Zadro et al [32], 2019	1	1	1	0	0	0	1	1	1	1	1	7	Good

^a^1: Eligibility criteria.

^b^2: random assignment.

^c^3: allocation concealment.

^d^4: baseline comparability.

^e^5: subject blinding.

^f^6: therapists’ blinding.

^g^7: assessor blinding.

^h^8: adequate follow-up.

^i^9: intention-to-treat analysis.

^j^10: comparisons between groups.

^k^11: point estimates and variability.

### Characteristics of the Included Studies

#### Study Characteristics

Seven included studies were published in years from 2011 to 2022. They were conducted in Spain [22], England [31], Canada [21,45], Brazil [44], Germany [46], and Australia [32]. Study designs were RCTs (n=4) [31,32,44,45], pilot RCTs (n=1) [46], and crossover pilot RCTs (n=2) [21,22]. The sample size ranged from 14 to 60, with a total of 264, of which 137 were in the intervention groups and 127 were in the control groups.

#### Participant Characteristics

All participants had musculoskeletal pain, participants in 5 studies [22,31,32,44,46] had chronic pain, and participants in the 2 studies [21,45] had nonchronic pain. The average age of the participants ranged from 67.8 (SD 6) years [32] to 81.85 (SD 6.82) years [22]. The majority of participants were female, accounting for around 69% of the total.

#### Intervention Characteristics

Interventions were conducted in the nursing home [22], the university’s physiotherapy laboratory [31], the hospital [45], the long-term care center [21], the center of rehabilitation [44], the laboratory [46], and the participant’s home [32]. The Nintendo Wii, the Active Airlines serious game, the Interactive Rehabilitation and Exercise System, and the ViRST VR application were the main gaming platforms in the experimental groups. For a period of 4 weeks to 8 weeks, participants exercised for 210 seconds to 90 minutes every session, only once during the intervention to 3 times per week. In 1 study, participants in the control groups carried on with their regular activities. The other studies used traditional physical therapy.

In 4 studies, participants performed exergaming under the supervision of the first author [31], therapist [21,45], and physiotherapist [46]. In 1 study [32], participants performed unsupervised home exergaming, and the remaining 2 studies did not report whether supervision was implemented [22,44]. The number of attrition people ranged from 1 to 9 due to personal commitments and fear of COVID-19 infection. Adherence was reported in only 3 studies [21,32,44].

Most of the participants in the experimental group had a positive experience. The occurrence of adverse events was not reported in 3 studies [31,44,45], while 3 studies reported no adverse events during the intervention [21,32,46]. Two individuals in 1 study experienced unpleasant symptoms such as dizziness, eye pain, or disorientation [22]. Multimedia Appendix 3 depicts an overview of the included study characteristics.

### Results of Studies

#### The Effect of Exergames on Pain

The complete pain data was presented in 7 papers. A study [22] comparing the effects of using Active Airlines serious game and conventional exercise on pain using Visual Analog Scale (VAS) measurements after 4 weeks discovered that both experimental and control groups had significant improvements in chronic neck pain, but exergaming therapy was not superior to conventional exercise. Ditchburn et al [31] compared exercise using the VR rehabilitation system to traditional gym-based exercise, and the results of the study, measured at baseline and after 6 weeks using the VAS, showed a significant improvement in chronic musculoskeletal pain intensity in the experimental group, but no significant change in the control group, and no statistically significant difference between the 2 groups. The difference in improvement in thermal pain, including burning and hot, measured by Multi Affect and Pain Survey, was significant between the 2 groups. In the study by Stamm et al [46], the results measured at baseline versus 6 weeks later on the Numeric Rating Scale (NRS) showed that the improvement in chronic back pain was not significant in the experimental group using the VR system and those in the control group receiving traditional pain therapy, and the difference between the 2 groups was not statistically significant. Fung et al [45] investigated the effect of Nintendo Wii Fit gaming sessions on pain in posttotal knee replacement individuals, finding no significant difference between the 2 groups when compared to a control group receiving lower extremity exercise. Two research investigated the effects of exergaming combined with traditional exercise with regular exercise on pain perception, with Nintendo Wii used. Hsu et al [21] used the NRS and the pain bothersomeness of the upper extremity to measure the improvement of pain in people with upper extremity dysfunction in the experimental and control groups after 4 weeks of treatment and found no significant differences. Another study [44] reported significant improvement in chronic low back pain after 8 weeks of treatment in participants in both the experimental and control groups using the NRS, however, the difference between the 2 groups was not statistically significant. The study by Zadro et al [32] evaluated the effect of home-based exercise via Wii Fit U on chronic low back pain, measured by NRS at baseline versus 8 weeks later, with no statistically significant difference compared to usual activities, however, after adjustment for baseline, the results showed a statistically significant difference between the 2 groups.

**Figure 4:**
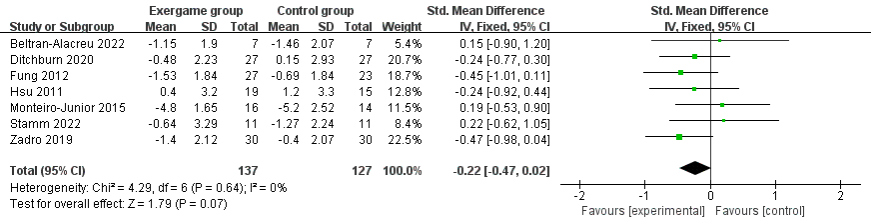
The effects of exergaming on pain perception.

A meta-analysis of data provided by VAS and NRS in 7 studies was performed and the overall results of this meta-analysis were inconclusive (SMD –0.22; 95% CI –0.47 to 0.02; *P*=.07; *I*^2^= 0%; the fixed-effect model; Figure 4).

#### Subgroup Analysis

**Figure 5:**
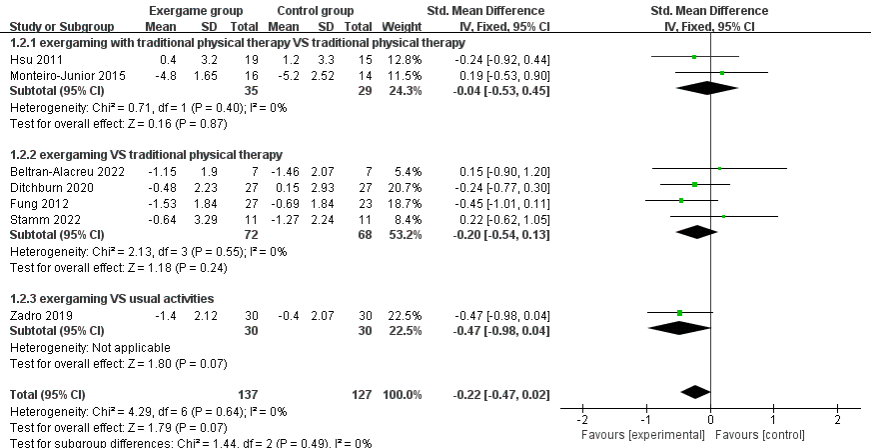
The effect on pain perception in the different the comparisons.

We investigated the differences in the effects of pain perception on the comparisons. Subgroup analysis showed no significant effect of exergaming in combination with traditional physical therapy (SMD –0.04; 95% CI –0.53 to 0.45; *P*=.87; *I*^2^= 0%; the fixed-effects model; Figure 5) or exergaming alone (SMD –0.2; 95% CI –0.54 to 0.13; *P*=.24; *I*^2^= 0%; the fixed-effects model; Figure 5) on pain perception compared to traditional physical therapy. There was no statistically significant difference between exergaming compared to usual activities (SMD –0.47; 95% CI –0.98 to 0.04; *P*=.07; *I*^2^= 0%; the fixed-effects model; Figure 5). The effect sizes for the 3 comparisons were small (<0.3), small (<0.3), and medium (≥0.3 and <0.6), respectively.

**Figure 6:**
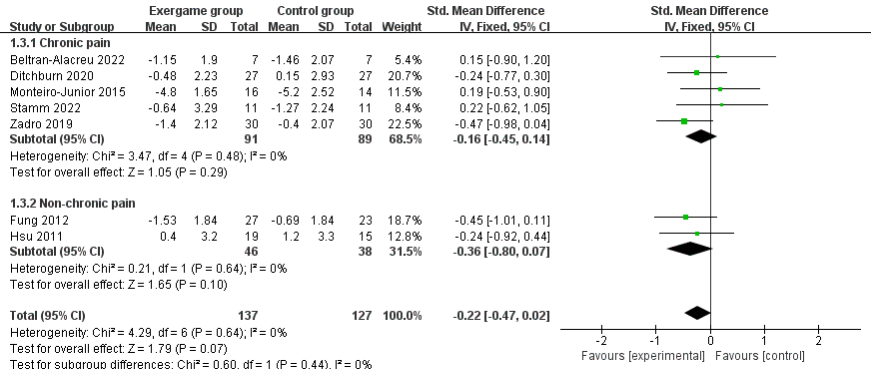
The effects of exergaming on chronic pain or nonchronic pain.

**Figure 7:**
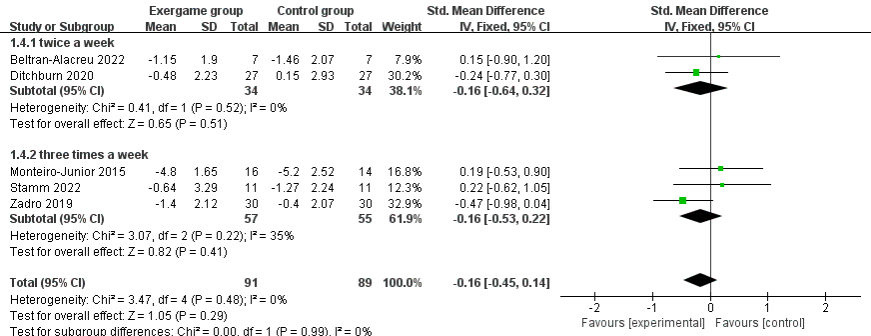
The effect of exercise frequency on chronic pain.

We conducted subgroup analyses to investigate the effects of exergaming on pain perception in participants with chronic pain or nonchronic pain. Subgroup analyses of the 7 studies revealed a nonsignificant difference in the effect of exergaming on chronic pain compared to controls (SMD –0.16; 95% CI –0.45 to 0.14; *P*=.29; *I*^2^= 0%; the fixed-effects model; Figure 6). Similarly, there was no statistically significant effect of exergaming on nonchronic pain compared to the control group (SMD –0.36; 95% CI –0.80 to 0.07; *P*=.10; *I*^2^= 0%; the fixed-effects model; Figure 6). Nevertheless, the effect sizes were small for chronic pain (<0.3) and moderate for nonchronic pain (≥0.3 and <0.6). We proceeded to investigate in depth the effect of exercise frequency on pain perception in participants with chronic pain. Subgroup analysis of the 5 studies showed no significant difference in the effect of exercise frequency of twice a week (SMD –0.16; 95% CI –0.64 to 0.32; *P*=.51; *I*^2^= 0%; the fixed-effects model; Figure 7) and 3 times a week (SMD –0.16; 95% CI –0.53 to 0.22; *P*=.41; *I*^2^= 35%; the fixed-effects model; Figure 7) on chronic pain compared to the control group, and the effect size was the same for both exercise frequencies (0.16).

## Discussion

### Efficacy

This meta-analysis and systematic review focused on the effects of exergaming on musculoskeletal pain in older adults and included 7 randomized controlled studies. The main finding is that the effect of exergaming on musculoskeletal pain in older adults is inconclusive. The results of this study are similar to Collado-Mateo et al [35], and they concluded that exergaming is more difficult to improve musculoskeletal pain in older individuals compared to adults. Of the 7 studies we included, 1 study [32] adjusted for baseline reported a significant improvement between groups. Another study [31] showed significant differences between the experimental and control groups in terms of improvement in thermal pain. Furthermore, the quality of the evidence is low and the sample sizes in the studies were quite small. Further research is needed on the effect of exergames on musculoskeletal pain in older adults.

The highest mean effect size of –0.47 was observed for improvement in pain in the comparison of exergaming versus usual activities, although it was not statistically significant. In this comparison, only 1 study [32] was included and adjusted for baseline, which showed statistical significance. This suggests that exergames have some potential in the treatment of musculoskeletal pain in older adults and that future high-quality studies are needed. Exergaming combined with traditional physiotherapy compared to traditional physiotherapy alone yielded the lowest mean effect size of –0.04, but still favored the experimental group despite the nonsignificant difference between the groups. The results of the 2 included studies were contradictory. Hsu et al [21] examined older adults with upper limb dysfunction, and after 4 weeks, the pain did not improve within either group. In contrast, in the study by Monteiro-Junior et al [44], the pain was significantly improved within both groups after 8 weeks, but the difference between the groups was not significant and showed results in favor of the control group. First, the difference in the results of the 2 studies may be related to the disease experienced by the participants, with different duration of the intervention. Second, the results of the study by Monteiro-Junior et al [44] favored the control group, possibly because participants in the experimental group had to complete not only traditional strength and core training, but also exergame training through the Nintendo Wii, with a total training time of 90 minutes each time, 3 times per week, and higher exercise intensity, which may have made people difficult to obtain optimal results. The results of the network meta-analysis by Fernández-Rodríguez et al [47] suggested that core exercises, strength exercises, or mind-body exercises for less than 60 minutes at a time, at least once to twice a week, with exercise lasting 3 to 9 weeks, are the most beneficial treatment for pain and disability in adults with chronic lower back pain exercise program. Due to older adults tend to have lower endurance levels, they are more susceptible to sports injuries and overexertion and have difficulty tolerating high-intensity training. The sample sizes of the 2 studies were small and the results should be interpreted with caution. In the comparison of exergaming with traditional physical therapy, the mean effect size was –0.2. The results favored the experimental group. Overall, exergaming can be used as adjunctive alternative therapy to traditional physical therapy.

These studies included older adults with a variety of musculoskeletal pain. Most of the participants in the studies suffered from chronic pain (*k*=5) such as back pain (*k*=3), neck pain (*k*=1), musculoskeletal pain (*k*=1), and other nonchronic pain such as upper extremity dysfunction (*k*=1) and post total knee replacement (*k*=1). These can be explained by the results of epidemiological studies, in which the most common pain complaints were osteoarthritic back pain, especially in the low back or neck (65%), musculoskeletal pain (40%), peripheral neuropathic pain (35%), and chronic joint pain (15%-25%) [9]. The results from the subgroup analysis showed that the effect sizes of exergaming on improving nonchronic pain were greater than the effect sizes on improving chronic pain. The results of this study are inconsistent with those of Collado-Mateo et al [35] and the results may be due to age-related group differences, and our study focused only on the group of older individuals. More studies are needed in the future.

The mechanisms by which exercise ameliorates pain are unclear, with 1 suggestion being that exercise leads to an increase in stress pain thresholds and that adaptation of central inhibition occurs over time with exercise training [48]. A meta-analysis showed that increasing the frequency of weekly exercise was most likely to have a positive impact on patients with chronic pain [49]. However, our study results showed that exercise frequency of twice a week and 3 times a week had the same size of effect on chronic pain. There are no standardized criteria for exergaming intervention programs, and it is particularly important to develop an appropriate exercise program. From the RCTs included in this study, 4 weeks of the exercise was sufficient to significantly improve pain, at least twice a week, but not for more than 90 minutes per session.

### Exergames Design

Most of the 7 studies tested commercial game platforms, with 1 study using a training rehabilitation-specific platform [31], and participants in the exergaming group experienced significant improvements in pain and for thermal pain, there was a significant difference between the 2 groups, the only 1 of the included studies to show a significant between-group difference for improvement in pain. Therefore, using professional rehabilitation exergames may be more effective than commercial games [23]. Professional exercise rehabilitation games are more specialized because they may be developed with the involvement of professionals in their design and can take into account the type of illness the users have, their needs, etc. Most commercial exergames are not suitable for the group of older individuals, about speed, required movements, amount of information, etc [50]. Therefore, in the future, commercial and medical rehabilitation professions should strengthen their cooperation to develop user-centered exergames for older individuals [51], thus improving the efficacy of exercise [52]. For older individuals, their physiological characteristics [53] and motivations for use should be considered. Older adults are motivated more by perceived health effects, the pleasure of the game, and the improvement of social confidence [20,54]. Wang et al [55] suggested that when designing exergames, first, aging characteristics should be included, paying attention to the decline of cognitive and physiological abilities associated with aging. Second, the game motion recognition should have higher fault tolerance. Third, the feedback should be clear. Fourth, consider the endurance of older individuals, pay attention to fatigue management and control the pace of the game. Fifth, it should have continuous action cues and tutorials. Sixth, it should be connected with reality. Seventh, reasonable use of body parts. Eighth, make good use of repetitive actions and reversible actions. Ninth, design advanced actions for the same game tasks. Lastly, designers should take advice from rehabilitation experts when designing exergames.

### Supervision and Adherence

Except for Zadro et al [32], who studied unsupervised home exergame training and found a significant effect of exergaming on pain after adjusting for baseline, the majority of research participants were supervised during exergaming. Compliance among participants was higher but still lower than with supervised exergame training. A prior study indicated that while home exercise training relieved low back pain, supervised training improved pain intensity the greatest [56]. However, to the best of our knowledge, no studies have been conducted to compare unsupervised home exergaming with unsupervised home exercise in pain relief for older individuals. Exergaming, in general, remains a highly promising kind of training that allows participants to undertake unsupervised therapeutic exercises at home, capable of generating a remote rehabilitation environment. Older individuals who are frail or incapacitated can obtain therapy without having to travel vast distances, which may have significant cost-effectiveness benefits [22]. Because just 1 study on home exercise was included in this paper, the results were insufficiently persuasive. As a result, further research might be done in the future, and methods to promote adherence to unsupervised exergame training in older people at home could be pursued.

### Security and Experience

Exergames are generally safe for older adults, although a few participants reported feeling uncomfortable, which may be related to the device and form of movement, such as wearing sensors to move the neck making participants feel dizzy, uncomfortable with their eyes, and disoriented [22]. From the reports of coaches and participants, it was found that exergames increased the fun and attraction of physical activity and made the game more enjoyable for the participants [21,45,46]. Previous research has also concluded that participants in the exergaming group had significantly more enjoyment of exercise than the other treatment groups [57]. Participants found exergames highly usable and the game challenging [22]. Exergames can increase participants’ satisfaction and compliance [22]. Some studies [20,46] considered socialization as an important factor in improving adherence, as stated in previous studies [58]. However, participants in a control group in 1 study showed a higher acceptance of traditional exercise than exergames [31], possibly because participants in the control group had not experienced exergames. Overall, older adults have positive attitudes toward exergaming.

### Limitations

It is the first systematic review of the efficacy of exergaming on musculoskeletal pain in older adults. Several limitations should be in consideration. When searching the literature, publications were limited to those in English and peer-reviewed ones. The small sample sizes of most studies were also a limitation. In addition, the overall risk of bias in the included studies was relatively high.

### Conclusions

This paper systematically reviews the efficacy of exergaming on musculoskeletal pain in older adults. The available evidence is limited, and therefore, exergaming cannot yet be considered an effective intervention for improving pain in older adults. Exergames are safe and cost-effective. The playfulness and social components of exergaming may contribute to participant adherence. Increased collaboration between industries to develop specialized exergames for older adults should be considered in the future. Overall, exergaming can be used as a complementary alternative to traditional training. Future larger sample sizes and rigorously designed RCTs are needed to explore the effects of different exergames on older adults with musculoskeletal pain.

## Appendix

Multimedia Appendix 1 Preferred Reporting Items for Systematic Reviews and Meta-Analyses (PRISMA) checklist.

Multimedia Appendix 2 Search strategy.

Multimedia Appendix 3 Characteristics of included studies.

## Abbreviations

NRS: Numeric Rating Scale
PRISMA: Preferred Reporting Items of Systematic Reviews and Meta-Analyses
RCT: randomized controlled trial
RoB 2: Revised Cochrane Risk of Bias tool in randomized trials
SMD: standardized mean difference
VAS: Visual Analog Scale
VR: virtual reality
