# Supplementary material for: Effects of Exergaming on Musculoskeletal Pain in Older Adults: Systematic Review and Meta-analysis
Source: JMIR Serious Games. 2023 Apr 25;11:e42944. doi: 10.2196/42944 (PMC10170365; doi:10.2196/42944)
Supplement: Multimedia Appendix 2 [file games_v11i1e42944_app2.docx]

**Multimedia appendix 1- search strategy**

| **Database** | **Search number** | **Search strategy** | **Number** |
| --- | --- | --- | --- |
| PubMed | #1 | ("Exergaming"[Mesh]) OR "Video Games"[Mesh] | 6572 |
|  | #2 | exergam* OR ((active video OR video OR computer OR serious OR motion OR interactive OR exercise) AND (game* OR gaming*)) OR virtual reality OR Wii OR Kinect | 46571 |
|  | #3 | #1 OR #2 | 46571 |
|  | #4 | aged OR elderly OR elder OR geriatric OR older OR senior | 6137139 |
|  | #5 | "Pain"[MeSH] | 429527 |
|  | #6 | pain* OR Ache* | 964309 |
|  | #7 | #5 OR #6 | 1044712 |
|  | #8 | #3 AND #4 AND #7 | 489 |
| CINAHL | S1 | AB (exergam* OR ((active video OR video OR computer OR serious OR motion OR interactive OR exercise) AND (game* OR gaming*)) OR virtual reality OR Wii OR Kinect) AND (pain* OR Ache*) AND (aged OR elderly OR elder OR geriatric OR older OR senior) | 153 |
| Cochrane Library | #1 | MeSH descriptor: [Exergaming] explode all trees | 4 |
|  | #2 | MeSH descriptor: [Video Games] explode all trees | 795 |
|  | #3 | (exergam*):ti,ab,kw OR ((active video OR video OR computer OR serious OR motion OR interactive OR exercise) AND( game* OR gaming*)):ti,ab,kw OR (virtual reality):ti,ab,kw OR (Wii):ti,ab,kw OR (Kinect):ti,ab,kw | 8736 |
|  | #4 | #1 OR #2 OR #3 | 8736 |
|  | #5 | MeSH descriptor: [Pain] explode all trees | 54054 |
|  | #6 | (pain*):ti,ab,kw OR (ache*):ti,ab,kw | 208203 |
|  | #7 | #5 OR #6 | 214567 |
|  | #8 | (aged OR elderly OR elder OR geriatric OR older OR senior):ti,ab,kw | 598780 |
|  | #9 | #4 AND #7 AND #8 | 355 |
| Web of  Science | #1 | TS=(exergam* OR ((active video OR video OR computer OR serious OR motion OR interactive OR exercise) AND (game* OR gaming*)) OR virtual reality OR Wii OR Kinect) AND (pain* OR Ache*) AND (aged OR elderly OR elder OR geriatric OR older OR senior) | 1088 |
| EMBASE | #1 | ‘exergame’/exp | 34 |
|  | #2 | ‘video game’/exp | 5032 |
|  | #3 | exergam*:ab,ti OR ((‘active video’:ab,ti OR video:ab,ti OR computer:ab,ti OR serious:ab,ti OR motion:ab,ti OR interactive:ab,ti OR exercise:ab,ti) AND (game*:ab,ti OR gaming*:ab,ti)) OR ‘virtual reality’:ab,ti OR Wii:ab,ti OR Kinect:ab,ti | 32999 |
|  | #4 | #1 OR #2 OR #3 | 34569 |
|  | #5 | aged:ab ti OR elderly:ab ti OR elder:ab ti OR geriatric:ab ti OR older:ab ti OR senior:ab ti | 1766859 |
|  | #6 | ‘pain’/exp | 1511714 |
|  | #7 | pain*:ab ti OR ache*:ab ti | 1176897 |
|  | #8 | #6 OR #7 | 1917553 |
|  | #9 | #4 AND #5 AND #8 | 283 |
