# Supplementary material for: Effects of Exergaming on Musculoskeletal Pain in Older Adults: Systematic Review and Meta-analysis
Source: JMIR Serious Games. 2023 Apr 25;11:e42944. doi: 10.2196/42944 (PMC10170365; doi:10.2196/42944)
Supplement: Multimedia Appendix 3 [file games_v11i1e42944_app3.docx]

**Multimedia Appendix 2-Characteristics of included studies**

| **Author/year/country** | **Design** | **Setting** | **Sample size(EG/CG)**  **Male/female** | **mean age**  **(EG/CG)** | **Physical**  **condition** | **Intervention group**  **(Systems/games)** | **Control group** | **Frequency and period of**  **the intervention** | **Attrition/**  **Supervision/**  **Adherence** | **Adverse**  **events** | **Experience** | **Measuring instrument** | **Result** |
| --- | --- | --- | --- | --- | --- | --- | --- | --- | --- | --- | --- | --- | --- |
| Beltran-Alacreu et al  2022  Spain | Randomized controlled  crossover  pilot trial | Nursing home | 14(7/7)  5/9 | 81.85±6.82 | Chronic neck  pain | **Active Airlines**  **serious game**  Control a virtual airplane to reach targets  using head motions | **Conventional exercise**  (Conventional physical  therapy with cervical  joint mobility exercises) | **EG:**  210s, twice a week, 4 weeks  **Conventional physical therapy:**  30 to 45 minutes, twice a week,  4 weeks  **Cervical joint mobility exercises:**  three sets of 12 repetitions of  cervical joint mobility exercises in allranges | No  NR  NR | Dizziness, eye  discomfort, ordisorientation | The global mean SEQ score was 50.38(SD 5.35) out of 65 points,  showing good suitability of the  serious game. Most patients  considered the experiencevery  enjoyable and “real” in the virtual environment and found the  information provided clear. Also,  they believed that the game could  be very helpful for their  rehabilitation. | VAS | VAS:  **A (P<.001)**  **B (P<.001)**  C (NS) |
| Ditchburn et al  2020  England | Randomized controlled  trial | University’s  physiotherapy laboratory | 54(27/27)  12/42 | 71.78±6.10/  69.78±4.48 | Chronic  musculoskeletal pain | **IREX^®^**  Volleyball, Sharkbait, Formula racing,  Snowboard, Birds and balls | **A traditional**  **gym-based exercise** | **EG:**  40-min, twice a week, 6 weeks  **CG:**  40-min, twice a week, 6 weeks | No  Supervised by the first  author  NR | NR | UTAUT scores increased in both  groups, indicating that participants  were moderately more receptive to  both forms of exercise. Significant  effects of the intervention were  found in favor of the control group in terms of social influence and  behavioral intentions. Higher means in the control group indicated a  higher acceptance of traditional  gym-based exercise than exergames. | NRS, MAPS | NRS:  **A (P<.01)**  B (NS)  C (NS)  MAPS (Thermal in  somatosensory pain):  A (NS)  B (NS)  **C (p＜.001)** |
| Fung et al  2012  Canada | Randomized controlled  trial | Outpatient  department  of a  rehabilitation hospital | 50(27/23)  17/33 | 67.9±9.5/  68.2±12.8 | Post total knee replacement | **Nintendo Wii Fit™ games +**  **physiotherapy session**  Ski Slalom, Tightrope Walk, Penguin Slide, Table Tilt,  Hula Hoop, Balance  Bubble, Deep Breathing, Half Moon, Torso Twist | **Lower extremity**  **exercises** (balance,  posture, weight shifting and strengthening  training) **+**  **physiotherapy session** | **Nintendo Wii Fit™ games:**  15 minutes, one session  **Lower extremity exercises:**  15 minutes, one session  **Physiotherapy session:**  60 minutes, one session | No  Supervised by treating  clinicians  NR | NR | There was no difference in satisfaction with treatment services between the two groups of participants  (p = 0.201). However, some  participants in the intervention  group expressed enjoyment of using the Wii Fit, such as ‘This is fun’  and ‘I’m glad I got to be in  this group’. No similar statements were noted within the control  group. | NRS | A (NR)  B (NR)  C (NS) |
| Hsu et al  2011  Canada | Randomized controlled  cross-over pilot trial | Long-term  care centre | 34(19/15)  10/24 | 80  (52 to 97) | Upper extremity dysfunction  (including pain,  weakness, and  stiffness in the  shoulder, elbow, wrist, or hand) | **Nintendo Wii bowling + CG**  bowling | **A standard exercise** | **Nintendo Wii bowling:**  a maximum of 20 minutes,  twice a week, 4 weeks  **Standard exercise:**  2 to 4 sessions a week | No  The trainers interacted withthe study participants in a standard way,  providing encouragementas appropriate  92% of the subjects completed at least six of the eight Wiis system treatments | No | Activity enjoyment measured with  the PACES-M reached statistical  significance (P=0.014). | NRS  Pain bothersomeness of the upper extremity | NRS:  A (NS)  B (NR)  C (NS)  Pain bothersomeness of the upper  extremity  A (NS)  B (NR)  C (NS) |
| Monteiro-Junior et al  2015  Brazil | Randomized controlled  trial | Rehabilitation center | 30(16/14)  0/30 | 68 ± 4 | Non-specific  chronic low  back pain | **Nintendo Wii-motion and**  **Wii Balance Board + CG**  Chair, Tightrope walk, Ski slalom, Balance  buble, Tabletilt,  Sideways, Rowing  squat, Lunge | **Strength exercises**  **and core training** | **EG:**  90 min, three times a week, 8 weeks  **CG:**  This training was consisted of  three sets of 15-30 seconds with afrequency of three times a week  during eight weeks | I=1, C=3,  (personal reasons)  NR  It is important to highlight a greater adhesion into EG (92%) than  CG (75%). | NR | unclear | NRS | **A (P<.01)**  **B (P<.01)**  C (NS) |
| Stamm et al  2022  Germany | Randomized controlled  pilot trial | Laboratory | 22(11/11)  8/14 | 75±5.80/  75.5±4.39 | Chronic back  pain | **ViRST VR**  **application**  Psychoeducational  sessions and perform  interactive taskson a  farm (marching,  rowing, balloon, pump,  hurdles, the bridge,  light bulbs, shaking bottles, ball bucket,  vegetable sorting,  apple tree) | **A conventional**  **multimodal pain**  **therapy** | **EG:**  30 min, three times a week, 4 weeks  **CG:**  30 min, three times a week, 4 weeks | I=9 (fear of covid  infection), C=0  Supervised by  physiotherapeutic  NR | No | The participants rated the VR  system as at minimum above  average on each individual subscale of the UEQ. The VR system  achieved an ‘excellent’ rating for  attractiveness and perspicuity.  Efficiency, dependability and  stimulation were rated as ‘good,’  while the originality of the VR  solution was rated as ‘above  average’. | NRS | NRS:  A (NS)  B (NS)  C (NS) |
| Zadro et al  2019  Australia | Randomized controlled  trial | Participants’  home | 60(30/30)  29/31 | 68.8±5.5/  67.8±6.0 | Chronic low  back pain | **Home-based Wii Fit U**  flexibility,  strengthening,  and aerobic exercises | **Usual activities** | **EG:**  60 min, three times a week, 8 weeks | I=1, C=2,  (personal commitments)  Unsupervised  home-based exercise  The mean (SD) total  exercise time (minutes) and number of sessions irrespective of duration was 1019.1 (489.5)  (70.8% of  recommendation) and  20.4 (9.3) (85.1% of  recommendation),  respectively. | No | Participants reported high usability  (average scores ranged from  7.9-8.7/10), sufficient exercise  variety (8.2/10) and challenge  (7.4/10), and a positive overall  experience using the program  (7.3/10). | NRS | A (NR)  B (NR)  Unadjusted C (NS)  **Adjusted C (P=.04)** |

**Notes:** EG, Experimental Group; CG, Control Group; SEQ, Suitability Evaluation Questionnaire; VAS, Visual Analog Scale; NS, No significant difference; IREX^®^, Interactive Rehabilitation and Exercise System; UTAUT, Unified Theory of Acceptance and Use of Technology; NRS, Numeric Rating Scale; MAPS, Multi Affect and Pain Survey; NR, not reported; PACES-M, Physical Activity Enjoyment Scale-Modified; UEQ, User Experience Questionnaire

A Intragroup differences in the EG relative to baseline

B intragroup differences in the CG relative to baseline

C Between-group differences relative to baseline
